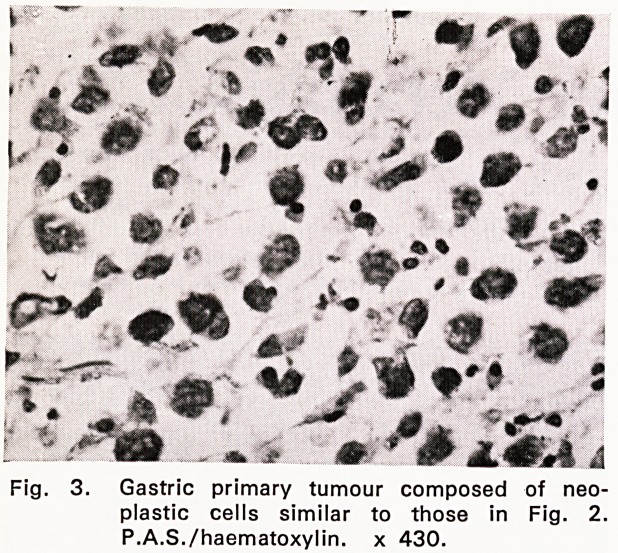# Tumour-To-Tumour Metastasis

**Published:** 1975-01

**Authors:** R. Salm

**Affiliations:** Royal Cornwall Hospital (Treliske), Truro, Cornwall


### Tumour-to-tumour Metastasis

R. SALM, M.D., F.R.C.Path.

Royal Cornwall Hospital (Treliske),
Truro, Cornwall

With the exception of metastatic involvement of adenomas of endocrine glands, metastasis of one primary tumour to another independent neoplasm is a rare occurrence. This paper records two such examples.

### CASE RECORDS

**Case 1.** M.J.C., female, aged 59 years, had a radical mastectomy for a scirrhous spheroidal-cell carcinoma in April 1959. The primary tumour measured 4 x 3.5 x 3.5 cm and one axillary lymphgland was invaded. Subsequently, an intra-abdominal mass arising from the pelvis was noted and in September 1959 a total hysterectomy and bilateral salpingoöophorectomy was performed. The patient died in March 1962, 3 years after mastectomy. There was no necropsy, the certified cause of death being widespread carcinomatosis.

**Operation specimen.** The uterus, tubes and left ovary were normal. The right ovary was considerably enlarged, 18 x 11 x 9 cm, and weighed 1250 g. It was firm and fibrous, with a smooth, slightly lobulated surface, and incorporated several reddish soft nodules up to 5 cm in diameter. Microscopical examination (59/1989) showed features typical of a Brenner tumour, with nodular metastases of scirrhous carcinoma, similar in appearance to the primary mammary growth (fig. 1).

**Case 2.** J.T., female, aged 63 years, was admitted to hospital because of abdominal enlargement and ascites. A total hysterectomy and bilateral salpingoöophorectomy was carried out in March 1960. She died 2 months later.

Fig. 1. Brenner tumour with thecomatous stroma invaded by solid carcinoma. Haematoxylin and eosin. x 110

Fig. 2. Endometrial polypus with dilated gland at left upper corner. Stroma invaded by spheroidal carcinomatous cells with dark-staining cytoplasm. Periodic acid-Schiff/haematoxylin. x 430

**Operation specimen.** There were bilateral ovarian Krukenberg tumours, 14 cm and 6 cm in diameter respectively. The uterine cavity was lined by atrophic endometrium containing a large sessile polypus. Microscopical examination (569/60) showed a senile endometrial polypus with cystically dilated glands. The stroma between the glandular acini was extensively invaded by spheroidal anaplastic epithelial cells with strongly PAS-positive cytoplasm (fig. 2). The adjacent endometrium was not involved.

**Necropsy** revealed a large gastric carcinoma with pleural and peritoneal metastases. Microscopical examination (1051/60) showed that the tumour was composed of anaplastic cells with PAS-positive cytoplasm similar to those seen in the endometrial polypus (fig. 3).

### DISCUSSION

Brenner tumours are uncommon. Hertig and Gore (1961) found an incidence of 1.7 per cent amongst all ovarian tumours. An incidence of 10.6 per cent was obtained by Biggart and Macaffee (1955) amongst tumours of the ovarian mesenchyme. On the other hand, ovarian metastases from a primary carcinoma of the breast are not rare. In the series of Willis (1973) the incidence was 6 per cent; in that of Symmers

Fig. 3. Gastric primary tumour composed of neoplastic cells similar to those in Fig. 2. P.A.S./haematoxylin. x 430.

(1966) 20 to 25 per cent; and Lumb and Mackenzie (1959) recorded an incidence of 63.3 per cent, although most of the secondary ovarian deposits demonstrated by the latter authors were of microscopical size. Spread of a mammary carcinoma to an ovarian Brenner tumour has so far not been recorded. It is interesting that in Case 1 metastases had developed in the Brenner tumour in spite of its very firm consistency, whilst the contralateral normal-textured ovary was not involved.

In case 2 the gastric carcinoma had produced an isolated uterine metastasis within the stroma of an endometrial polypus. It might be questioned whether an endometrial polypus should be regarded as a benign tumour, many authors regarding such polypi as areas of focal endometrial hyperplasia. However, Bird and Willis (1970) considered endometrial polypi to be true neoplasms, representing the benign counterpart of malignant mixed endometrial tumours (or carcinosarcomas).

Two or more different tumours coexisting in the same individual have been noted by many authors (Jernstrom and Murray, 1966), but tumour-to-tumour metastases are rare; they can conveniently be subdivided into four subgroups.

**(1) Metastases to adenomas of endocrine glands**
In Willis's (1973) series of 21 cases of metastatic involvement of the thyroid gland, metastases were present within the adenomatous areas in 7 cases. He also noted a similar preference of malignant involvement for cortical adenomas of the adrenal gland, and Woolner, Keating and Black (1958) reported a metastasis from a breast carcinoma in a parathyroid adenoma. Amongst 41 benign tumours containing metastastes reviewed by Ortega, Li and Shimkin (1951) there were 15 thyroid adenomas, 10 cortical adrenal adenomas and 1 parathyroid adenoma, which supports Willis's (1973) view that adenomas of endocrine glands consitute a favourable soil for metastatic growth.

**(2) Metastases to lymphomatous foci**
Most case reports of this type have been concerned with carcinomatous spread to lymphomatous regional lymph nodes. However, such cases cannot be regarded as genuine examples of tumour-to-tumour metastasis, for lymphatic spread of a carcinoma is bound to result in carcinomatous deposits in the regional lymph nodes, irrespective of whether these are lymphomatous or not.

Haematogenous carcinomatous metastases to coexisting widespread lymphoma are an entirely different matter. Rabson et al. (1954, Case 1) reported one such patient, showing blood-borne deposits from a caecal adenocarcinoma in lymphosarcomatous areas of lungs, liver and pancreas.

**(3) Metastases to benign host tumours**
Such cases are comparatively rare. Ortega et al. (1951) and Willis (1967) reported metastases from breast carcinomas to uterine leiomyomas, and Jackson and Symmers (1951) recorded a deposit from a rectal adenocarcinoma in a squamous papilloma of the skin. Wheelock, Frable and Urnes (1962, case 18) mentioned a metastatic breast carcinoma involving an endometrial polypus; Willis (1973) referred to a similar case, and case 2 in the present paper is a further example. Other benign tumours acting as hosts to malignant deposits are meningiomas and neurofibromas (Willis, 1973); and Berg (1955) reported a bronchial carcinoma metastasizing to a renal angiolipomyoma.

Cystic ovaries have occasionally been the seat of metastatic tumour deposits. Ley (1919), Taylor (1929) and Wechsler (1926) mentioned this occurrence briefly, and Küster (1911) reported and depicted metastases from a gastric carcinoma involving bilateral combined cystadenomas/dermoid cysts of ovary. Jackson and Symmers (1951, case 3) reported a secondary deposit from a breast carcinoma in an ovary which had been largely replaced by a dermoid cyst. This, however, was a secondary deposit in the ovarian remnant which had involved the dermoid cyst only secondarily.

**(4) Metastases to malignant host tumours**
According to Gore and Barr (1958) hypernephromas have been the recipient malignant tumours in two-thirds of all cases. The metastasizing tumours have mostly been bronchial carcinomas and, to a lesser extent, carcinomas of the breast, prostate, stomach, endometrium and thyroid (Dobbing, 1958; Gore and Barr, 1958). An ocular melanoma metastasizing to an hypernephroma was reported by Ortega et al. (1951). Comparatively often have hypernephromas been found to be the host tumours because microscopically the usually darkly staining invading neoplasms have been conspicuous against the background of the large clear cells of the hypernephroma. Similarly, as reported by Towers (1961), bronchial oat-cell carcinomas invading prostatic adenocarcinomas contrast sharply with the tissues of the host tumour. Conversely, if both the invading and the recipient tumours are similar in histological appearances, definite proof of tumour-to-tumour metastasis cannot usually be furnished.

### SUMMARY

Two examples of tumour-to-tumour metastasis are reported: a breast carcinoma metastasizing to an ovarian Brenner tumour, and a gastric carcinoma metastasizing to a senile endometrial polypus. The findings are discussed together with those of previously recorded cases.

I am greatly indebted to Drs. T. K. Owen and D. J. Parish for the data and material of case 2, and to Dr. G. I. Barrow for scrutinizing the manuscript. My thanks are also due to Mr. W. M. Seymour, Chief Technician, for his skilled technical and photographic assistance.

### REFERENCES

BERG, J. W. (1955) **Cancer, 8,** 759. Angiolipomyxosarcoma of kidney (malignant hamartomatous angiolipomyoma) in a case with solitary metastasis from bronchogenic carcinoma.

BIGGART, J. H. AND MACAFEE, C. H. G. (1955) **Journal of Obstetrics and Gynaecology of the British Empire, 62,** 829. Tumours of the ovarian mesenchyme.

BIRD, C. C. AND WILLIS, R. A. (1970) **British Journal of Cancer, 24,** 759. The possible carcinogenic effects of radiations on the uterus.

DOBBING, J. (1958) **Guy's Hospital Reports, 107,** 60. Cancer to cancer.

GORE, I. AND BARR, R. (1958) **Archives of Pathology, 66,** 293. Metastasis of cancer to cancer.

HERTIG, A. T. AND GORE, Hazel. (1961) Tumours of the female sex organs. Atlas of tumor pathology, Fascicle 33, pt.3, p.124. Washington D.C.: Armed Forces Institute of Pathology.

JACKSON, J. G., AND SYMMERS, W. St.C. (1951) **British Journal of Cancer, 5,** 38. Coexistence at one site of two neoplasms, one of local origin and one metastatic.

JERNSTROM, P., AND MURRAY, G. C. (1966) **Cancer, 19,** 60. Synchronous double primary lymphosarcoma and adenocarcinoma (collision tumour) of the stomach with cancer-to-cancer metastasis.

KÜSTER, H. (1911) **Zeitschrift für Geburtshilfe und Gynäkologie, 68,** 364. Zur Histologie der metastatischen Ovarialkarzinome.

LEY, G. (1919-20). **Proceedings of the Royal Society of Medicine, 13,** 95. Primary and secondary carcinoma of the ovary: A statistical record from the pathological institute of the London Hospital.

LUMB, G., AND MACKENZIE, D. H. (1959) **Cancer, 12,** 521. The incidence of metastases in adrenal glands and ovaries removed for carcinoma of the breast.

ORTEGA, P., LI, Irene Y., AND SHIMKIN, M. (1951) **Annals of Western Medicine and Surgery, 5,** 601. Metastasis of neoplasms to other neoplasms.

RABSON, S. M., STIER, P. L., BAUMGARTNER, Geraldine C., AND ROSENBAUM, D. (1954) **American Journal of Clinical Pathology, 24,** 572. Metastatis of cancer to cancer.

SYMMERS, W. St.C. (1966) **The Breasts in Systemic Pathology,** Ed. G. P. Wright and W. St.C. Symmers, p.1010. London: Longmans.

TAYLOR, H. C. (1929) **Surgery, Gynaecology and Obstetrics, 48,** 204. Malignant and Semimalignant tomours of the ovary.

TOWERS, R. P. (1961) **Journal of the Irish Medical Association, 48.** 79. Unusual metastatic behaviour of double primary tumours of prostate and lung.

WECHSLER, H. F. (1926) **Archives of Pathology, 2,** 161. Primary carcinoma of the Fallopian tubes.

WHEELOCK, M. C., FRABLE, W. J., AND URNES, P. D. (1962) **American Journal of Pathology, 37,** 475. Bizarre metastases from malignant neoplasms.

WILLIS, R. A. (1967) **Pathology of tumors,** 4th edition, **p.243.** London: Butterworth.

WILLIS, R. A. (1973) **The spread of tumours in the human body,** 3rd edition, pp. 198, 221, 270, 294. London: Butterworth.

WOOLNER, L. B., KEATING, F. R., AND BLACK, B. M. (1958) **Cancer, 11,** 975. Primary hyperparathyroidism and breast carcinoma: A case in which breast carcinoma metastasized to a parathyroid adenoma.

## Figures and Tables

**Fig. 1. f1:**
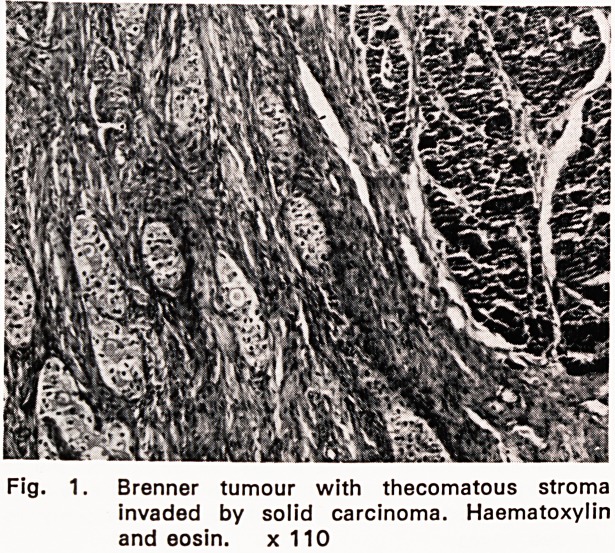


**Fig. 2. f2:**
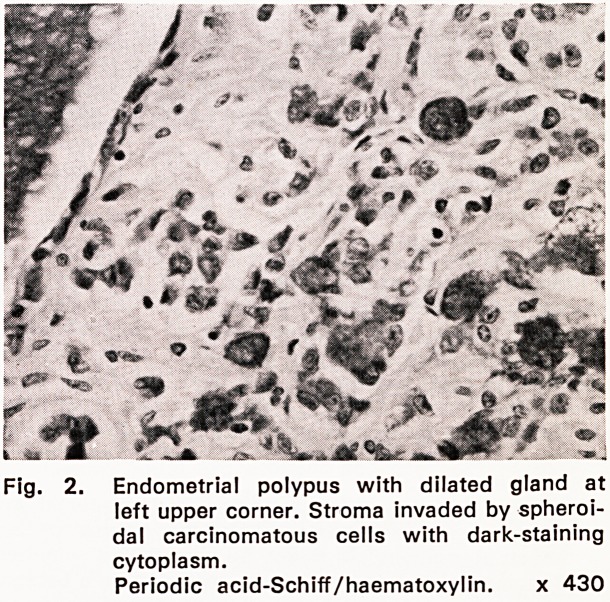


**Fig. 3. f3:**